# Pathophysiology and Clinical Utility of Non-coding RNAs in Epilepsy

**DOI:** 10.3389/fnmol.2017.00249

**Published:** 2017-08-10

**Authors:** Yiye Shao, Yinghui Chen

**Affiliations:** ^1^Department of Neurology, Jinshan Hospital, Fudan University Shanghai, China; ^2^Department of Neurology, Shanghai Medical College, Fudan University Shanghai, China

**Keywords:** non-coding RNA, epilepsy, microRNA, long non-coding RNA, circular RNA

## Abstract

Epilepsy is a common neurologic disorder. The underlying pathological processes include synaptic strength, inflammation, ion channels, and apoptosis. Acting as epigenetic factors, non-coding RNAs (ncRNAs) participate in the regulation of pathophysiologic processes of epilepsy and are dysregulated during epileptogenesis. Aberrant expression of ncRNAs are observed in epilepsy patients and animal models of epilepsy. Furthermore, ncRNAs might also be used as biomarkers for diagnosis and the prognosis of treatment response in epilepsy. In this review, we will summarize the role of ncRNAs in the pathophysiology of epilepsy and the putative utilization of ncRNAs as diagnostic biomarkers and therapeutic targets.

Epilepsy is a chronic neurologic disorder which is characterized by recurring unprovoked seizures and affects more than 50 million people worldwide. In China, the prevalence rate is around 2.89%, and 40–50% of these patients do not receive the necessary treatment. China has more than nine million existing cases and about 45,000 new patients suffering from epilepsy each year (Gu et al., 2013). Epilepsy entails recurrent seizures, an increased mortality rate, a huge economic burden, and decreased quality of life. At present, the main treatment is antiepileptic drugs (AEDs), but ~30% of epilepsy patients do not respond to AEDs, a condition known as refractory epilepsy. None of the AEDs currently in use focus on the pathogenesis of epilepsy. Therefore, it is necessary to search for new ways to treat epilepsy. Currently, the pathogenesis of epilepsy is not completely clear. A growing number of studies have shown that ncRNAs participate in pathological and physiological processes in the nervous system, for instance the development of the nervous system, synaptic plasticity, learning and memory, oxidative stress and so on (Aksoy-Aksel et al., 2014; Nwaobi et al., 2014; Karnati et al., 2015; Loke et al., 2015). These characteristics suggest that ncRNAs may play an important role in the pathogenesis of epilepsy. NcRNAs refer to small endogenous RNAs that do not code for proteins, or function without being translated into a protein. Only 1.5% of the human genome appears to code for proteins; about 80% of the rest of the genome transcribes into ncRNAs (Huang et al., 2016). A number of studies have shown that ncRNAs play important roles in epigenetic modifications (Molina, 2017; Redis and Calin, 2017). NcRNAs are divided into many subtypes such as rRNA (ribosomal RNA), tRNA (transfer RNA), snRNAs (small nuclear RNA), snoRNA (small nucleolar RNA), miRNA (microRNA), siRNA (small interfering RNA), piRNA (piwi-interacting RNA), eRNA (enhancer associated RNA), lncRNA (long non-coding RNA), and circRNA (circular RNA) (Redis and Calin, 2017). In this review, we will highlight recent studies examining the latest advances in ncRNAs and their relations with epilepsy.

## MicroRNA and epilepsy

miRNA is a class of small non-coding RNA (ncRNA) which participates in post-transcriptional regulation. The primary transcript is first produced in the nucleus and then processed into hairpin RNA (pre-miRNA) by the Drosha microprocessor complex. Exportin-5 transfers pre-miRNA out of the nucleus for its final processing into mature miRNA by the RNAase III enzyme Dicer (O'Carroll and Schaefer, 2013). Mature miRNA forms RNA-induced silencing complex (RISC) with Argonaute (Ago) proteins, which can form sequence-specific base-pairings with the target mRNA, usually with the 3′ untranslated region (UTR) (Chandradoss et al., 2015; Lin and Gregory, 2015). This formation can lead to mRNA degradation or translational inhibition and then regulates the expression of target genes. Recent studies have demonstrated that miRNA can also regulate promoters with CpG island methylation at the transcriptional level (Benhamed et al., 2012; Tan et al., 2013).

### Aberrant expression of miRNAs in epilepsy

Important roles for miRNA in brain development have been observed, as well as roles in tissue-specific expression in the brain. The aberrant expression of miRNAs was observed in the blood and brain tissues of animal models of epilepsy and epileptic patients (Table 1; Wang et al., 2015b; Zhu et al., 2015). Studies have demonstrated that these aberrant expressions of miRNAs link to the mechanisms of epileptogenesis through regulating ion channels, neuronal morphology, synaptic plasticity, inflammatory response, and neuronal apoptosis. Through comparing the sera miRNA expression of 117 epilepsy patients and 112 healthy controls, it has been found that let-7d-5p, miR-106b-5p, miR-130a-3p, and miR-146a-5p were up-regulated in the sera of epilepsy patients, whereas miR-15a-5p and miR-194-5p were down-regulated (Wang et al., 2015a,b). Another study found that the expression of miR-34a, miR-22, miR-125a, and miR-21 in blood and the hippocampus were changed 24 h after the onset of status epilepticus (SE) (Hu et al., 2011). Roncon et al. (2015) detected miRNA expression in the hippocampal granule cell layer and in plasma at a different phase of the development of pilocarpine-induced epileptic rats. Sixty-three differentially expressed miRNAs were found in the granular cell layer (GCL). When validating miRNAs which were up-regulated in the chronic phase of epileptic rats in brain tissues from epileptic patients, they found that miR-21-5p, miR-23a-5p, miR-146a-5p, and miR-181c-5p were also up-regulated in human brain tissues. They also found that 27 miRNAs were differentially expressed in the plasma samples of epileptic patients. Chak et al. (2016) reported that miR-21 is up-regulated in epilepsy and that pre-miR-21 may attenuate miR-21-mediated suppression following SE and could potentially lead to prolonged TGF-β receptor expression, which can impact epileptogenesis. Gorter et al. (2014) detected miRNA expression in the CA1, dentate gyrus, and parahippocampal cortex regions after electrically-induced SE at 1 day, 1 week, and 3–4 months in a rat model for temporal lobe epilepsy. The expression of miRNAs exhibited dynamic changes after SE and changed differently in different regions. In CA1, 18 miRNAs were up at 1 day, 16 at 1 week, and 7 in the chronic phase. In dentate gyrus, 20 miRNAs were up at 1 day, 15 at 1 week, and 37 at 3 months after SE. In parahippocampal cortex, 31 miRNAs were up at 1 day, 37 at 1 week, and 22 in the chronic stage.

**Table 1:** miRNAs profiling in epilepsy models and patients.

**Tissues**	**Dysregulated miRNAs**	**Regulation**	**Pathways targeted by the miRNAs**	**References**
Sera of epilepsy patients	let-7d-5p, miR-106b-5p, -130a-3p, -146a-5p	Up	Regulating oxidative responses, inflammation responses and immune responses through interleukin 1β and cell adhesion molecules (miR-146a)	Wang et al., 2015b
	miR-15a-5p, -194-5p	Down		
Hippocampus and blood of status epilepticus rats	miR-213, -132, -30c, -26a, -375, -99a, -24, -124a, -22, -34a,-125a, -101-1, -29b, -125b, -199a, -196b, -150, -151, -145	Up	miR-134 altered the number and volume of dendritic spines through Limk1 miR-132 has a neuroprotective effect through the miR-132/p250GAP/Cdc42 pathway	Wang et al., 2015a
	miR-29a, -181c, -215, -181b, -25, -10b, -21	Down		
Hippocampal granule cell layer of pilocarpine-induced epileptic rats	miR-15b-5p, -17-5p, -18a-5p, -19a-3p, -19b-3p, -20a-5p, -20b-5p, -21-5p, -23b-5p, -24-3p, -27a-3p, -92a-3p, -93-5p, -142-3p, -344b-2-3p, -431,-466b-5p, -674-3p,-129-1-3p, -129-2-3p, -129-5p, -181c-5p, -181d-5p, -409a-5p, -655, -874-3p	Up	miR-296-5p regulate apoptosis through targeting caspase-3	Hu et al., 2011
	miR-7a-1-3p, -107-3p, -138-5p,-139-3p, -186-5p, -204-5p, -222-3p, -324-3p, -505-3p, -296-5p, miR-500-3p and miR-652-3p	Down		
Plasma of pilocarpine-induced epileptic rats	miR-466b-1-3p, -494-3p, -598-5p, -32-3p, -300-3p, -30c-2-3p, -101b-3p, -142-3p, -142-5p, -181a-1-3p, -374-5p, -466c-3p, -1188-3p, -3065-3p, -3582	Down		Hu et al., 2011
Hippocampal granule cell layer of epilepsy patients	miR-21-5p, -23a-5p, -146a-5p, -181c-5p	Up		Chak et al., 2016
Hippocampal of status epilepticus mice	miR-132, -219, -323, -21, -507, -518d	Up	Silencing miR-132 has a neuroprotective effect through the miR-132/p250GAP/Cdc42 pathway	Gorter et al., 2014
	miR-657, -520b	Down		
Sera of epilepsy patients	miR-106b, -146a, -301a	Up	miR-146a can regulate inflammatory response	Wang et al., 2015a
	miR-194-5p	Down		
Sera of mesial temporal lobe epilepsy patients	miR-143-3p, -145-3p, -365a-3p, -532-5p	Up		Weber et al., 2010
Sera of epilepsy patients	miR-574-5p, -67, novel-9, -144-5p, -15a-5p, -181c-5p, -194-5p, -889-3p, -96	Up		Wang et al., 2015b
	let-7d-5p, -106b-5p, -130a-3p, -146a-5p, -194-5p, -204-5p, -221-5p, -301a-3p, -30b-5p, -342-5p, -3605-5p, -4446-3p, -598-3p, -874-3p, -889-3p, novel-451	Down		

Araujo et al. (2016) performed a miRNA microarray of the hippocampi of Wistar rats 24 h after intra-hippocampal pilocarpine-induced SE. A total of 73 miRNAs were found to be significantly dysregulated, of which 36 were up-regulated and 37 were down-regulated. Among these miRNAs, the expression level of miR-352 and 196b-5p were over-expressed and miR-128a-3p were under-expressed. They also evaluated the three miRNAs at three time points: 0 h, 24 h and chronic phase after SE. They found that the expression of miR-128a-3p was significantly down-regulated at the three time points compared to the control group, miR-352 was significantly up-regulated at 24 h post-SE and in chronic phase, while miR-196b-5p was significantly upregulated only at 24 h post-SE. They found that the expression levels of miRNAs show similar trends to the rat models when compared with the hippocampi of epileptic patients with control group.

miR-134 is a brain-specific miRNA, and the high expression of miR-134 was observed after the occurrence of epilepsy and SE. Silencing of miR-134 expression could reduce the density of the CA3 pyramidal neuron dendrite spine in the hippocampus and suppress the injury caused by seizures and prolonged seizures. Morphometric analysis of dendritic spines revealed that miR-134 could increase neuron volume and decrease spine volume (Jimenez-Mateos et al., 2012; Wang et al., 2014). It has been demonstrated that LIM kinase-1 (Limk1) plays important roles in dendritic spine morphogenesis through phosphorylating and inactivating cofilin (Meng et al., 2002). Loss of Limk1 results in abnormal spine morphology. Studies found that miR-134 inhibits LIM kinase-1 (Limk1) mRNA, thus preventing protein translation of Limk1. Pretreatment of mice with miR-134 antagomirs before pilocarpine administration reduced the number of mice that developed SE, attenuated the degree of epileptic seizures and increased survival rate (Jimenez-Mateos et al., 2015). In addition, other studies observed expression of miR-134 decreased after the occurrence of epilepsy. miR-134 may prevent synaptic plasticity by inhibiting CREB and p-CREB expressions, thereby exerting neuroprotective qualities (Zhu et al., 2015).

miR-128 is abundant in the brains of humans and adult mice, and the expression of miR-128 increases gradually during the process of growth, peaking in adulthood. miR-128 is encoded by miR-128-1 and miR-128-2. miR-128-2 plays a major role. miR-128-2^−/−^ mice progress rapidly to fatal epilepsy and death. The high expression of miR-128 in mice can reduce seizures through suppressing neuronal excitability and abnormal motor activity. Studies suggest that miR-128 may function through suppressing the dopamine 1 receptor, which can increase neuronal excitability and abnormal motor activity (Tan et al., 2013). Yuan et al. (2016a) reported that miR-128 was down-regulated in glioblastoma, and knockout of the miR-128a could induce epilepsy in a mouse model. Furthermore, dysregulation of miR-128 expression may be associated with glioma-associated epilepsy in low-grade glioma.

miR-146a has been widely studied in the inflammatory response. High expression of miR-146a was observed in both epileptic animal models and epilepsy patients (Matos et al., 2014; Roncon et al., 2015). Studies found that miR-146a expression was at its lowest level at acute seizure phase and at its highest level at the latent phase. Another study revealed that expression of miR-146a was increased in the chronic phase of epileptic rats and remained at high levels 1 week after SE (Gorter et al., 2014; He et al., 2016). Functional polymorphisms of the gene miR-146a may also play a role in the regulation of epilepsy. Rs2910164 and rs57095329 are two SNPs in the gene miR-146a, which can modify the expression level of mature miR-146a. Studies found that rs57095329 may be associated with drug resistance and frequency of seizures (Cui et al., 2015).

Li et al. (2016) found that miR-153 was down expressed in plasma and temporal cortexes resected from surgical mTLE patients compared with control patients. miR-153 might down-regulate HIF-1α expression through binding with two sites in the 3′UTR region of HIF-1α transcript. miR-153 may serve as a putative regulator of hypoxia-inducible factor-1α (HIF-1α), which participates in multidrug resistance in refractory epilepsy. Functional experiments showed that miR-153 mimics can inhibit HIF-1α expression in a pharmacoresistant astrocyte model. miR-153 may regulate the expression of HIF-1α in mTLE and serve as a putative biomarker and treatment target for epilepsy.

Yuan et al. (2016) induced epileptic neurons by exposing the neurons to magnesium-free medium for 3 h, which were considered as a useful *in vitro* model of refractory SE. They found that silencing miR-132 has a neuroprotective effect on cultured epileptic neurons, including inhibiting the electrical excitability level of cultured epileptic neurons. Using a lithium-pilocarpine-induced epileptic mouse model, they found that silencing miR-132 can inhibit the aberrant formation of dendritic spines and chronic spontaneous seizures. Another study found that pretreatment with miR-132 antagomirs can reduce hippocampus injuries after SE. Upregulation of miR-132 may be associated with neuronal death (Jimenez-Mateos et al., 2011).

### miRNAs and neurogenesis in epilepsy

Neurogenesis impairment participate in the pathophysiology of epilepsy in humans and also observed in animal models (Mendonca et al., 2017). Aberrant hippocampal neurogenesis can cause epilepsy and seizures can also effect hippocampal neurogenesis. Activation, migration, integration of neural stem cells, and expression of brain plasticity-associated proteins in hippocampus may be required for the maintenance of the kindling criterion (Retchkiman et al., 1996; Schmoll et al., 2003; Buga et al., 2012; Uemori et al., 2017). More and more evidence suggests that miRNAs participate in neurogenesis, which play an important role in the pathophysiological mechanism of epilepsy. miR-124 has been reported to regulate Neuron Restrictive Silencer Factor (NRSF). NRSF was widely expressed in neural stem cells, and regulates the differentiation, diversity and plasticity of neural stem cells. NRSF can also repress the genes HCN1 and KCC2, which can regulate neural activity through ion channels (Brennan et al., 2016). Franzoni et al. (2015) found that miR-128 participates in neuronal migration and intrinsic excitability. miR-146a was reported to participate in the epileptogenesis and progression of seizures through tGCLhe regulation of inflammation and immune responses (He et al., 2016).

### Pathways targeted by miRNAs

**Figure 1:**
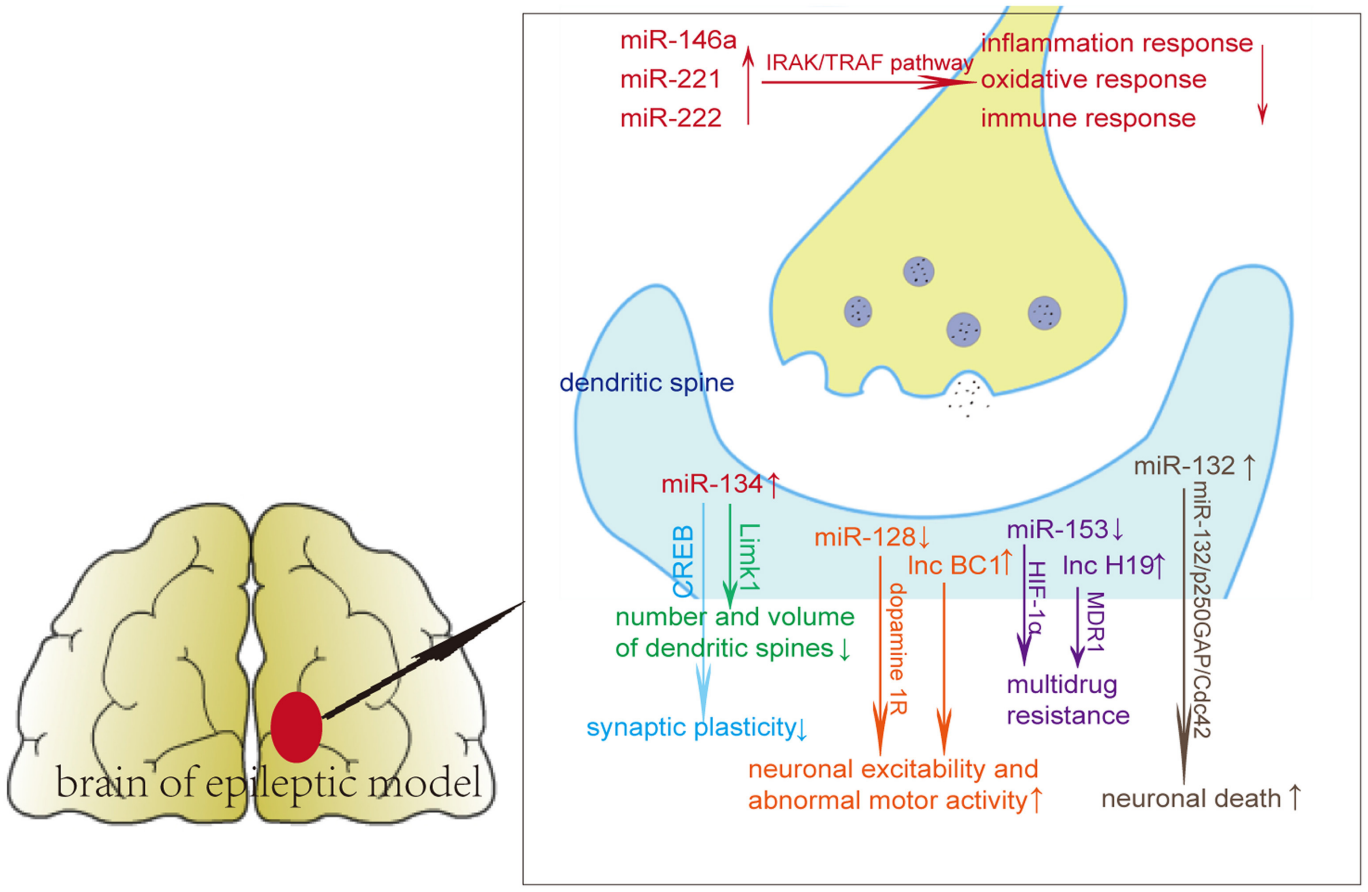
Potential pathways of non-coding RNAs in epileptic brain tissues.

A series of functional studies found that miRNAs affect seizures via affecting neuroinflammation or apoptosis. An individual miRNA can have different targets, regulating single genes in several pathways or several genes in a single pathway (Ebert and Sharp, 2012). The dysregulation of miRNAs probably affects various molecular and cellular pathways in epilepsy, including inflammation, oxidative stress, immune responses, axon guidance, cell differentiation, migration, and proliferation (Figure 1, Table 1). miR-134 regulates the number and volume of dendritic spines, presumably through its target Limk1, which plays important roles in dendritic spine morphogenesis through phosphorylating and inactivating cofilin (Jimenez-Mateos et al., 2015). miR-146a, miR-221, and miR-222 can participate in epilepsy via regulating oxidative responses, inflammation responses and immune responses through targets such as interleukin 1β and cell adhesion molecules (Aronica et al., 2010; Kan et al., 2012).

Yan et al. (2016) found that the potential target genes of miR-3613-5p, miR-4668-5p, miR-8071, miR-197-5p, miR-4322, and miR-6781-5p, predicted by bioinformatics analysis, were involved in biological processes, molecular functions, and cellular components through affecting the calcium signaling pathway, the MAPK signaling pathway and the PI3K-Akt signaling pathway. These miRNAs may regulate seizure development in mesial temporal lobe epilepsy with hippocampal sclerosis (mTLE-HS). Yuan et al. (2016) found that silencing miR-132 has a neuroprotective effect on cultured epileptic neurons and lithium-pilocarpine-induced epileptic mouse models through the miR-132/p250GAP/Cdc42 pathway by regulating the morphology and electrophysiology of dendritic spines. Over-expression of miR-184 was observed in mesial temporal lobe epilepsy patients with hippocampal sclerosis compared with mesial temporal lobe epilepsy patients without hippocampal sclerosis. miR-184 may regulate inflammatory responses through regulating inflammatory signal transduction and apoptosis (Danis et al., 2016).

### miRNAs as putative biomarkers of epilepsy

A series of studies have reported that changes of miRNAs observed in certain biological fluids correlate with various pathological conditions suggesting that circulating miRNAs might be useful and informative biomarkers to reflect the pathological status of the body (Valentino et al., 2017; Wang, 2017; Zendjabil et al., 2017; Zhao et al., 2017). Evidence has emerged that miRNAs in blood or cerebrospinal fluid may serve as potential biomarkers of brain injury (Weber et al., 2010; Kulstein et al., 2016; Martinez and Peplow, 2016; Sirker et al., 2016; Wang, 2017). These miRNAs in biological fluids may come through the damaged blood–brain barrier after epilepsy onset or originate from controlled release in exosomes (Choi et al., 2017; Gourlay et al., 2017). These miRNAs circulate in blood or cerebrospinal fluid after release due to encapsulation in extracellular vesicles or complexing with proteins (Turchinovich et al., 2012). Animal studies in epileptic rat models suggest that specific miRNA in blood plasma and sera reflected different types of brain injury, including dynamic change after seizure onset and a pattern unique to prolonged seizures (Gorter et al., 2014; Yan et al., 2016).

Wang et al. (2015b) found that miR-106b-5p was up-regulated in sera from epilepsy patients compared with healthy controls, and miR-106b-5p had 80.3% sensitivity and 81.2% specificity in diagnosis of epilepsy. Roncon et al. (2015) reported that the alterations of miR-9a-3p, miR-466b-1-3p, miR-494-3p, and miR-598-5p occurred earlier than the onset of the first spontaneous seizure in a rat model. These miRNAs may be proposed as putative biomarkers of epileptogenesis. Gorter et al. (2014) compared the expression levels of miR-21-5p, miR-146-5p, and 142-5p in plasma with brain tissue in as SE rat model. They found that the expression pattern of miR-21-5p at different time points was similar to that observed in brain tissue. However, the pattern of expression of miR-146a-5p and 142-5p was different from the pattern observed in brain areas. miR-21-5p may serve as a potential biomarker in plasma to reflect dynamic changes in brain tissue. Sun et al. (2016) found that miR-30a was upregulated in the sera of epileptic patients and the expression level of miR-30a was positively associated with seizure frequency at the onset of seizures. Of a total of 50 microRNAs, 2 were increased and 48 were decreased and found to be differentially expressed in mTLE-HS compared with healthy controls. Among these miRNAs, miR-8071 had 83.33% sensitivity and 96.67% specificity in mTLE-HS diagnoses and was associated with seizure severity (Yan et al., 2016). Surges et al. (2016) found that more than 200 miRNAs were differentially expressed within 30 min of seizure onset in the sera of patients. Among these miRNAs, miR-143, miR-145, miR-532, and miR-365a were significantly deregulated. They also found 10 miRNAs to be differentially expressed 20–28 h after seizures in patients with seizures occurring during sleep. miR-663b was significantly deregulated among these 10 miRNAs. Detectable transient miRNA alterations in blood sera were detected after single seizures in the early postictal phase. Li et al. (2016) found that miR-153 was down-regulated in temporal cortexes resected from surgical mTLE patients compared with control patients. Down-regulation of miR-153 in the plasma of mTLE patients was also observed in an independent validation cohort. These studies suggest that the expression level of miRNAs in blood or cerebrospinal fluid is deregulated after epilepsy onset, and some may relate to the severity and frequency of epilepsy. Furthermore, the expression of these miRNAs circulate in biofluids are stable. Due to these characteristics miRNAs may serve as potential biomarkers of epilepsy in the future.

### Prospects for miRNAs therapeutics

At present, diagnosis of epilepsy is mainly based on the clinical symptoms, neuroimaging and electroencephalograms. Early diagnosis is closely related to the clinical prognosis of epilepsy patients. miRNAs may be used as a biomarker in the early diagnosis of epilepsy due to its tissue-specific and stable expression. However, the multi-targeting and multi-pathway actions of miRNAs make prediction and therapeutic function difficult. Several studies have reported the potential therapeutic targets of miRNAs; these studies provide a novel prospect of epilepsy treatment and have improved our understanding of the targets of these miRNAs. A series of functional studies have reported the potential therapeutic targets of epilepsy on miRNA levels through administration of mimics or antagomirs (Jimenez-Mateos et al., 2012; Wang et al., 2014; Yuan et al., 2016). Pre-administration of miR-134 antagomirs to pilocarpine mice reduced the number of mice that triggered SE, attenuated the seizure degree of epileptic mice and increased survival rate (Jimenez-Mateos et al., 2015). Yuan et al. (2016a) reported that dysregulation of miR-128 expression may participate in glioma-associated epilepsy in low-grade glioma, and that knockout of the miR-128a could induce epilepsy in mouse models. Yuan et al. (2016) found that silencing miR-132 decreased the electrical excitability level in epileptic neurons, inhibited the aberrant formation of dendritic spines and attenuated chronic spontaneous seizures in a lithium-pilocarpine-induced epileptic mouse models. Upregulation of miR-132 may be associated with neuronal death and pretreatment with miR-132 antagomirs can attenuate hippocampus injuries after SE (Jimenez-Mateos et al., 2011). These results suggest that silencing miR-132 has a neuroprotective effect and that miR-132 may serve as a putative target for developing antiepileptic treatment.

Further studies focusing on the effect of miRNAs in animal models or epileptic patients are needed in the future.

## Long non-coding RNA

Long non-coding RNAs (lncRNAs) are transcripts longer than 200 nucleotides that have little or no protein-coding capacity, and have drawn intense research efforts recently (Schaukowitch and Kim, 2014). While lncRNAs were at first considered transcriptional noises, recent studies have demonstrated that they play essential roles in biological pathways, including X inactivation, imprinting, development, and differentiation (Danis et al., 2016; Valentino et al., 2017; Zendjabil et al., 2017). lncRNAs can regulate many processes in mammalian and gene expression through a diversity of mechanisms and at different levels, but the available research tools are still insufficient. Therefore, their functions in epilepsy remain unclear. It is known that lncRNAs can serve as molecular scaffolds for combinations of chromatins and proteins, such as homeobox A1 (HOXA1). lncRNAs can also act as enhancers, for instance as enhancer RNAs (eRNAs). They can also repress transcription through inhibiting RNA polymerase II or mediating chromatin remodeling and histone modification (Hewson et al., 2016).

Lee et al. (2015) performed a microarray analysis to compare lncRNAs expression in pilocarpine and kainate models with control mice to study epileptic mechanisms. They found 384 aberrant lncRNAs in the pilocarpine model and 279 aberrant lncRNAs in the kainate model. These dysregulated lncRNAs may participate in epileptic mechanisms. lncRNAs may regulate the occurrence and development of epilepsy through a diversity of mechanisms, such as neurogenesis, regulation of neurotransmitter, ion channels, and synaptic plasticity (Ng et al., 2013). Xiao et al. (2017) reported that aberrantly methylated lncRNA and pathway targets might be involved in TLE development and progression. Abnormal development of the nervous system may cause epilepsy. lncRNAs participate in embryonic morphogenesis and neuronal differentiation in the embryonic period (Mercer et al., 2010). For example, Dlx1as can modulate the expression of neighboring homeobox genes, thereby regulating neuronal differentiation (Ramos et al., 2013). Evf2 plays a role in the regulation of homeodomain transcription factors and the formation of GABA-dependent neuronal circuitry in the developing mouse forebrain (Bond et al., 2009). lncRNAs also participate in synaptic plasticity. lncRNA Malat1 can increase the density of dendritic spines and thereby modulate synaptic plasticity and neuronal regeneration (Wu et al., 2013). The lncRNAs BC1 and BC200 modulate protein synthesis in postsynaptic dendritic microdomains and are thought to play roles in signal transduction and synaptic plasticity. Studies have found that deficiency of BC1 increases neuronal excitability and facilitates the progression of epilepsy (Gitaí et al., 2011). Studies on cancer have found that lncRNA H19 increases multidrug resistance 1 (MDR1) expression and MDR1-associated drug resistance in liver cancer cells through regulating MDR1 promoter methylation. It is considered that MDR1 also plays important roles in the drug resistance of refractory epilepsy. It may be supposed, therefore, that lncRNAs may also participate in refractory epilepsy.

## Circular RNA

CircRNAs are a novel type of noncoding RNA differing from linear RNAs. A covalent bond linking the 3′ and 5′ ends of circRNA form closed loop structures (Jeck et al., 2013; Chen et al., 2015). Nuclease hydrolyzes target the tails of linear RNAs, while circular RNAs form closed loop structures; therefore, circRNAs are not susceptible to degradation by RNA exonuclease or RNase (Chen et al., 2016). Due to the stability of circRNAs, expression of circRNAs is more abundant than the corresponding linear mRNAs in plasma (Li Y. et al., 2015; Qin et al., 2016; Shao and Chen, 2016). CircRNAs are highly enriched in eukaryotic organisms and display elevated sequence conservation with specific expression in various tissues during different developmental stages (Memczak et al., 2013; Tan et al., 2017). CircRNAs can act as competitive endogenous RNAs (ceRNAs); the temporal and spatial specificity of circRNA expression supports this possibility (Dong et al., 2016; Ebbesen et al., 2016; Gruner et al., 2016; Szabo et al., 2016). Some circRNAs contain miRNA response elements (MREs) and can interact with miRNAs as miRNA sponges (Hansen et al., 2013; Li F. et al., 2015). Recently gathered evidence indicates that circRNAs participate in the micro-regulation of miRNA expression levels (Zhao et al., 2016; Xue et al., 2017). CircRNAs can perturb miRNA inhibitory functions on target mRNAs by competitive binding with miRNAs, and they can then regulate the expression of target genes (Peng et al., 2016; Zhao et al., 2016; Tang et al., 2017). For instance, sex-determining region Y (SRY) can act as a natural miRNA sponge to suppress miR-138 activity (Yeh et al., 2013; Granados-Riveron and Aquino-Jarquin, 2014; Zhao and Shen, 2015). CircRNAs can modulate the expression of other RNAs through partial base pairing with target RNAs. For example, CDR1as can increase CDR1 mRNA stability through complementary base pairing with CDR1 mRNA. CircRNA can recruit the components of multiprotein complexes or directly combine with proteins and regulate their activity. For example, CDR1as can bind with the protein Argonaute (AGO). Recent studies have found that circRNAs can act as translation templates to encode proteins (Xu et al., 2015; Geng et al., 2016).

CircRNAs may participate in diseases through competitive binding with target miRNAs based on their characteristics (Yu et al., 2016; Xu et al., 2017). Many studies have demonstrated that aberrant circRNAs are associated with the initiation and development of various diseases, but much of the effort in these studies into circRNAs has initially been devoted to cancer research (Zhao and Shen, 2015; Nair et al., 2016; Li et al., 2017). The relationship with epilepsy has not been reported, but the regulatory role of circRNAs is considered widespread. Studies have found that circRNAs are abundant in brain tissues and have specific distribution (Dong et al., 2016; Kumar et al., 2016). For instance, circRNAs are highly expressed in neuropils, especially in dendrites, and participate in regulating synaptic function (You et al., 2015). This suggests that circRNAs may participate in the regulation of synaptic function and neuroplasticity, which play an important role in the development of epilepsy. The antisense to the cerebellar degeneration-related protein1 transcript (CDR1as) acts as a natural miRNA sponge, and can negatively regulate miR-7. High CDR1as expression can decrease miR-7 activity by binding to it and thus increasing target gene expression (Geng et al., 2016). miR-7 is an important regulatory factor in signaling pathways, and it can regulate many other regulatory factors such as epidermal growth factor receptor (EGFR), insulin receptor substrate-1 (IRS-1), AKT3, IRS-2,UBE2A, CTGF, p21-activated kinase- 1 (Pak1), and Raf1(Reddy et al., 2008; Peng et al., 2016; Zhao et al., 2016; Tang et al., 2017). Both CDR1as and miR-7 are abundant in brain tissues, and these regulatory factors play important roles in epilepsy. Thus, we speculate that circRNAs may participate in the epileptogenesis and development of epilepsy. Studies have reported that some circRNAs seem to have virus miRNA binding sites and can affect immune responses. Wang et al. (2015) reported that circular RNA100783 may be involved in chronic CD28-associated CD8 (+) T cell aging and global immunosenescence. miR-138 has a protective effect on brain injury and can regulate T helper 1 (Th1) and T helper 2 (Th2) expressions via inhibiting the function of runt-related transcription factor 3 (RUNX3) and regulate IL-1β through targeting Forkhead Box C1 (Fu et al., 2015; Tang et al., 2016; Yuan et al., 2016b). Circular RNA SRY can act as natural miRNA sponges to suppress miR-138 activity (Granados-Riveron and Aquino-Jarquin, 2014). The above studies suggest that circRNA may participate in inflammatory reactions that induce neuropathy, therefore participating in the epileptogenesis and development of epilepsy.

## Conclusions

There are still significant gaps in our current understanding of ncRNAs compared with coding RNAs, and many functions remain unclear. Due to the abundance and stability of ncRNAs in circulating fluids, they may be considered as clinical diagnosis biomarkers in the future. Further study of ncRNAs will improve our understanding regarding epileptogenesis and pathogenesis of epilepsy and lead to new methods of diagnosis and treatment.

## Author contributions

YS conceived the content and wrote the critical review. YC provided the ideas and supervised the work. Both authors read and approved the final version of the manuscript.

### Conflict of interest statement

The authors declare that the research was conducted in the absence of any commercial or financial relationships that could be construed as a potential conflict of interest.
